# From Biomarkers to Models in the Changing Landscape of Chronic Lymphocytic Leukemia: Evolve or Become Extinct

**DOI:** 10.3390/cancers13081782

**Published:** 2021-04-08

**Authors:** Isabel González-Gascón-y-Marín, Carolina Muñoz-Novas, Ana-Eugenia Rodríguez-Vicente, Miguel Quijada-Álamo, María Hernández-Sánchez, Claudia Pérez-Carretero, Victoria Ramos-Ascanio, José-Ángel Hernández-Rivas

**Affiliations:** 1 Department of Hematology, Hospital Universitario Infanta Leonor, 28031 Madrid, Spain; carolinacecilia.munoz@salud.madrid.org (C.M.-N.); vramosa@salud.madrid.org (V.R.-A.); jahernandezr@salud.madrid.org (J.-Á.H.-R.); 2 Centro de Investigación del Cáncer, Department of Hematology, Hospital Universitario de Salamanca, Salamanca-CSIC University, IBSAL, IBMCC, 37007 Salamanca, Spain; anita82@usal.es (A.-E.R.-V.); mquijada@usal.es (M.Q.-Á.); mariahs@usal.es (M.H.-S.); claupeca@usal.es (C.P.-C.); 3 Facultad de Medicina, Universidad Complutense de Madrid, 28040 Madrid, Spain

**Keywords:** chronic lymphocytic leukemia, prognosis, targeted therapy

## Abstract

**Simple Summary:**

Chronic lymphocytic leukemia (CLL) is characterized by a highly variable clinical course. Thus, predicting the outcome of patients with this disease is a topic of special interest. The rapidly changing treatment landscape of CLL has questioned the value of classical biomarkers and prognostic models. Herein we examine the current state-of-the-art of prognostic and predictive biomarkers in the setting of new oral targeted agents with special focus on the most controversial findings over the last years. We also discuss the available information on the role of “old” and “new” prognostic models in the era of oral small molecules.

**Abstract:**

Chronic lymphocytic leukemia (CLL) is an extremely heterogeneous disease. With the advent of oral targeted agents (Tas) the treatment of CLL has undergone a revolution, which has been accompanied by an improvement in patient’s survival and quality of life. This paradigm shift also affects the value of prognostic and predictive biomarkers and prognostic models, most of them inherited from the chemoimmunotherapy era but with a different behavior with Tas. This review discusses: (i) the role of the most relevant prognostic and predictive biomarkers in the setting of Tas; and (ii) the validity of classic and new scoring systems in the context of Tas. In addition, a critical point of view about predictive biomarkers with special emphasis on 11q deletion, novel resistance mutations, TP53 abnormalities, IGHV mutational status, complex karyotype and *NOTCH1* mutations is stated. We also go over prognostic models in early stage CLL such as IPS-E. Finally, we provide an overview of the applicability of the CLL-IPI for patients treated with Tas, as well as the emergence of new models, generated with data from patients treated with Tas.

## 1. Introduction

Chronic lymphocytic leukemia (CLL) is the most frequent chronic leukemia in Western countries. The diagnosis is usually incidental in a routine blood test and its outcome is extremely heterogeneous. Some patients present with a rapidly progressive evolution, while others remain at an indolent state for the rest of their lives. Antitumor therapy is only required if active disease is documented, according to the International Workshop on Chronic Lymphocytic Leukemia (iwCLL) criteria [1]. Furthermore, response to treatment is also variable and may be predicted by different biomarkers. This is of vital importance at this time, in which treatment algorithms have drastically changed and chemoimmunotherapy (CIT) has been replaced by targeted agents (Tas) for most patients [2,3]. Research is moving ahead at a staggering speed and, consequently, the therapeutic arsenal is growing. Oral targeted treatments approved and available worldwide are: ibrutinib, the first generation of Bruton Tirosine Kinase inhibitors (BTKi); idelalisib, the first generation of phosphatidyl-inositol 3-kinase inhibitors (PI3Ki); and venetoclax (BCL-2 inhibitor). The European Medicine Agency (EMA) has just approved acalabrutinib, a second class BTKi. The second class PI3Ki, duvelisib is also available in some countries. Other second class BTKi (zanubrutinib), PI3Ki (umbralisib) or new reversible, non-covalent BTKi (pirtobrutinib, ARQ 531) are under investigation and will hopefully be available soon [4,5,6,7,8,9,10,11,12,13,14,15,16,17,18]. Therefore, the identification of prognostic and predictive biomarkers is relevant, not only for patient counseling but also for planning follow-up or selecting treatment at a time where a shift towards personalized medicine is taking place. Table S1 summarizes the names and principal characteristics of clinical trials with TAs and CIT in CLL mentioned throughout this review.

The difference between the terms prognostic and predictive biomarker has been previously addressed in depth [19,20]. In brief, prognostic biomarkers separate groups of patients with different outcomes regardless of treatment. On the contrary, a predictive biomarker provides information about the possible benefit of a specific treatment and can be used in the clinical decision-making process [21]. Many of the most powerful prognostic and predictive biomarkers were identified in the CIT era [22,23,24,25,26,27,28] but the validity of most of them has been evaluated also with oral Tas [6,11,12,29,30,31].

Although individual factors can be a very important prognostic tool, reality is more complex, as each patient may harbor several biomarkers with different prognostic value. To overcome this issue, prognostic scores have been developed integrating biomarkers into models. The Rai and Binet systems, proposed almost half a century ago, were the pioneers and, despite their limitations, they are still in force today [32,33]. Since then, various prognostic models and nomograms were proposed that can be applied at different moments during the course of the disease. The most established today is the CLL-International Prognostic Index (CLL-IPI), which has demonstrated its ability to predict overall survival (OS), time to first therapy (TTFT) and progression-free survival (PFS) in the CIT setting [34,35]. It has also shown to predict TTFT in early-stage CLL [36] and community-based cohorts of patients [37,38]. However, its utility to predict PFS and even OS in patients treated with Tas is limited [39]. Thus, other models have recently emerged to evaluate prognosis in this setting [40,41].

Herein, we discuss data evaluating the usefulness of prognostic and predictive biomarkers for patients treated with Tas. We also focus on novel prognostic models and the value of conventional models for patients treated with Tas.

## 2. Prognostic Biomarkers: All That Glitters Is Not Gold

**Figure 1:**
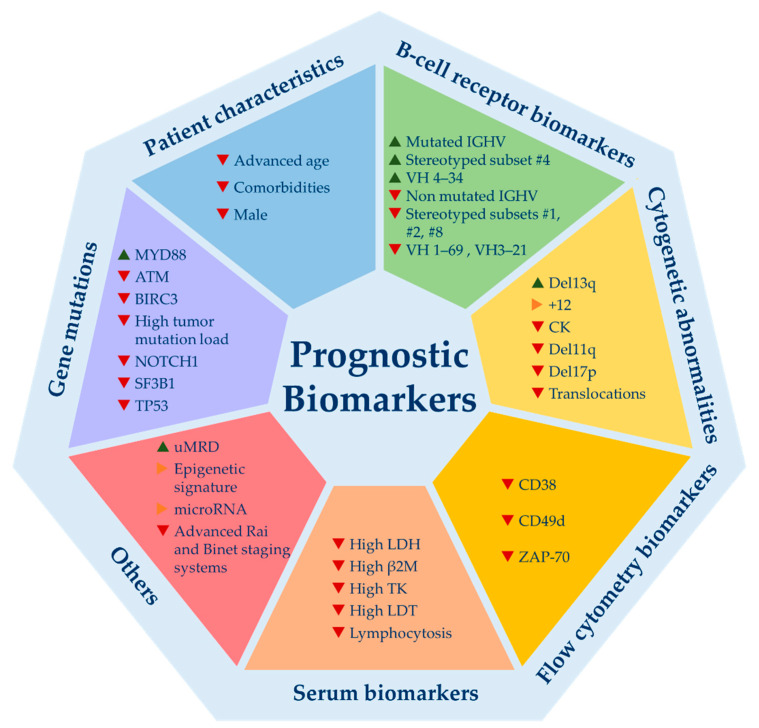
Relevant prognostic biomarkers for chronic lymphocytic leukemia. Del13q = 13q deletion; +12 = trisomy 12; CK = complex karyotype; del11q = 11q deletion; del17p = 17p deletion; LDH = lactate dehydrogenase levels; β2M = beta-2-microglobulin levels; TK = thymidine kinase; LDT = lymphocyte doubling time; uMRD = undetectable minimal residual disease. ▲ indicates good prognosis; ▶ indicates good and bad prognosis or intermediate prognosis; ▼ indicates poor prognosis.

Over the last 50 years, plenty of biomarkers with ability to predict CLL evolution were identified. The most relevant, classified by categories, are illustrated in Figure 1.

Even though they emerged in the era of CIT, most are valid today, since they are capable to predict time to first treatment (TTFT), which is not influenced by the choice of therapy [22,23,24,25,42,43,44,45,46,47,48,49,50,51,52,53,54,55,56,57,58,59,60,61,62,63,64,65]. The mutational status of immunoglobulin heavy chain variable region (IGHV) gene, cytogenetic abnormalities detected by FISH, CD49d expression and *TP53* mutations are the biomarkers that have been consolidated as the most powerful ones and are supported by the best scientific evidence [50,66]. Others such as ZAP-70 and CD-38 have lost their strength, although their prognostic value is unquestionable. These flow cytometry biomarkers may be useful if IGHV mutation status is not available, as they act as surrogate markers. Among B-cell receptor biomarkers, a selective usage of IGHV genes in CLL has been described, with an overuse of certain genes. Some of these gene usages have been associated with clinical outcome such as VH1-69, VH3-21 (bad outcome) or VH 4-34 (good outcome) [67,68,69,70]. In addition, almost a third of CLL patients express stereotyped B cell receptor immunoglobulins (BcR IG). Some of these subsets also harbor prognostic value highlighting subsets #1, #2, #8 (bad prognosis) and #4 (good prognosis) [63,71]. Recently, a single point mutation in IGLV3-21 (R110-mutated IGLV3-21) has been studied, identifying an aggressive biological subtype of CLL [72].

Recurrent gene mutations identified by next generation whole exome or whole genome sequencing carry important prognostic information [61,73,74]. However, its implementation in routine practice has not been fully recommended to date, with the exception of *TP53* mutation [1]. A great variety of mutations have been identified, but only a few occur in more than ~5% of the patients. Among them stand out *NOTCH1*, *SF3B1*, *ATM*, *BIRC3*, *POT1* and *MYD88*. All but *MYD88* have been associated with adverse outcome and other poor prognostic biomarkers [75]. Some patient-related and tumor-load variables such as age, comorbidities, beta-2-microglobulin levels (B2M), lymphocytosis or lymphocyte doubling time (LDT) are available in virtually all patients and remain valid in predicting TTFT [76,77]. Novel markers such as complex karyotype (CK), stereotyped subsets, micro-RNAs or epigenetic subsets need more evidence to be used in the routine setting. Finally, minimal residual disease (MRD) is one of the strongest predictors of PFS and OS in CLL patients treated with CIT [78]. Indeed, undetectable MRD (uMRD) is considered a surrogate marker for PFS in the context of clinical trials. Regarding targeted treatments, BTKi or PI3Ki obtain very long PFS despite their low rates of complete responses (CR) and uMRD. Therefore, uMRD is not a valid prognostic biomarker for patients treated with BTK or PI3K inhibitors [5,6,11,12,13]. Conversely, venetoclax-based regimens induce high rates of uMRD enabling a fixed-duration treatment which has established uMRD as a therapeutic goal for these combinations. Moreover, the prognostic value of achieving uMRD with venetoclax has been demonstrated, not only in the relapsed/refractory (R/R) setting but also as a frontline treatment (Murano and CLL14 phase 3 trials) [10,31]. Combos of novel agents (TA) between them +/− anti-CD20 monoclonal antibodies or, less frequently, with CIT is what immediate future holds. Preliminary results of trials using these combinations are impressive with the highest rates of uMRD ever seen (>50–70%), which might turn uMRD as the most powerful biomarker to predict prognosis in CLL patients that require treatment [79,80,81,82,83]. In fact, it could be used to guide treatment decisions in the near future by helping to decide when to stop or intensify therapy. Nevertheless, questions such as how to proceed with MRD results after a fixed duration schedule (stop, continue or change treatment) remain open. In summary, despite this large amount of biomarkers, not all have been externally and prospectively validated and, furthermore, few are valuable for clinical decision-making.

## 3. Predictive Biomarkers in the Targeted Therapy Era: Something Old, Something New, Something Borrowed and Something Blue

Predictive biomarkers allow anticipating the response to a specific treatment. In fact, they constitute the cornerstone on which therapeutic algorithms are based. As the therapeutic armamentarium expands, the need to identify and validate predictive biomarkers is reinforced with the aim of offering the treatment that best suits each patient. Nowadays, American and European guidelines base their treatment algorithms on age and comorbidities, IGHV mutation status and the presence of *TP53* mutation or del(17p) [2,3]. In summary, Tas are recommended in all settings as first line treatment, highlighting their preferred use over CIT for patients with *TP53* abnormalities (*TP53* mutations or del(17p)) or unmutated IGHV status. The exception where CIT might be appropriated, at the same level as Tas, is for young patients without *TP53* abnormalities and mutated IGHV status. This is justified by the results of the ECOG-ACRIN E1912 clinical trial that compared ibrutinib-rituximab versus fludarabine-cyclophosphamide-rituximab (FCR) in patients 70 years of age or younger. The combination of ibrutinib-rituximab resulted to be superior to FCR in terms of PFS and OS. This benefit was observed for all subgroups with the exception of IGHV-mutated patients, in which both treatments achieved similar results and a long follow-up is required to determine the best option for this population. [8]. Given the change in the therapeutic algorithm that has occurred, some predictive factors will lose their value, especially if CIT disappears from the treatment scenario. Others are emerging to help tailoring treatment decisions that involve Tas and combinations. In this section, we summarize the current situation of factors with predictive value with special focus on BTKi (ibrutinib, acalabrutinib) and BCL-2 inhibitors (venetoclax).

### 3.1. Something Old: Invalid Biomarkers for Current Treatment Algorithms That Were Important Previously and Might Reappear

#### 11q Deletion

Twenty years ago, Döhner et al. demonstrated the prognostic value of certain cytogenetic abnormalities detected by FISH. The 11q deletion (del(11q)) is detected in around 20% of the patients who require first line treatment and is associated with unfavorable outcome [42,84]. Initial studies with CIT pointed out that the addition of anti-CD20 could overcome the bad prognosis that del(11q) entailed, suggesting that this abnormality could be considered a predictive biomarker for increased response to FCR or obinutuzumab-chlorambucil (O-Chl) [85,86]. Later, as antiCD-20 was added universally to chemotherapy protocols, its predictive utility disappeared. More than 80% of the patients with del(11q) have an unmutated-IGHV (U-CLL) pattern, another poor outcome marker for patients treated with CIT. In the low proportion of patients with mutated-IGHV (M-CLL) and del(11q), the prognostic role that del(11q) adds is controversial. The CLL-8 trial found that this subgroup of patients (M-CLL and del(11q)) had an excellent outcome, similar to other M-CLL [87]. In contrast, results from an Italian multicenter retrospective study with 404 patients, showed that M-CLL patients without del(11q) had a better outcome than those who carried this deletion [88].

The prognostic value of del(11q) in patients treated with ibrutinib has recently been addressed in a pooled analysis that compiled results from three phase 3 clinical trials that recruited 620 patients. In this study, a longer PFS and a possible OS benefit (not significant) was observed for patients with del(11q) who were treated with ibrutinib compared to those without this cytogenetic alteration [89]. Indeed, the authors suggested that del(11q) could be used as a predictive biomarker for better outcomes in patients receiving this drug. Anyway, this observation needs further validation and studies with preclinical models harboring this alteration, which will help to understand 11q-related effects on treatment response [90]. Direct comparison with other treatment options such as venetoclax or acalabrutinib combinations should be also explored. In fact, acalabrutinib and venetoclax also overcome the bad prognosis of del(11q), as reported in the subgroup analysis of major phase 3 trials of both molecules [11,12,30,31].

### 3.2. Something New: Novel Biomarkers for the Tas Treatments

#### 3.2.1. Resistance Mutations to BTK Inhibitors

Despite the excellent results provided by ibrutinib, a few patients do not respond (primary resistance) and others relapse during treatment (acquired resistance). A responsible mutation can be identified in around 60–80% of the patients with acquired resistance [91,92,93]. These mutations occur at the binding site of ibrutinib to BTK, usually at position C481S. Less frequent, activating mutations in the PLCG2 pathway might be detected. In some patients, both types coexist [91,92,94]. Interestingly, *BTK* and *PLCG2* mutations were not observed in patients without previous exposure to ibrutinib and usually appear between the second and fourth year under treatment [91]. Not only that, some studies were able to detect these mutations in samples around 9–15 months before relapse happened [95]. Nevertheless, it is unknown whether all patients with treatment mutations will relapse, and how long it will take. In conclusion, acquired mutations could represent biomarkers of resistance to ibrutinib. The advantage of their early detection and the possible switch to other treatments is still to be determined. Recent reports have shown that mechanisms of resistance to acalabrutinib are similar than to ibrutinib, which is not surprising as acalabrutinib uses the same position (C481S) to bind to BTK [96]. For readers interested in this topic, comprehensive reviews have been published and are available in the references cited below [97,98,99,100,101].

#### 3.2.2. BCL-2 Mutations

Resistance to venetoclax is a more complex process in which different independent molecular mechanisms are involved [102]. However, a mutation at the G101V location in BCL-2 has been found but in around 50% of the patients in a small study that analyzed samples from 15 patients relapsing on venetoclax. This mutation behaves similar to BTK C481S on three aspects: it affects the venetoclax affinity to BCL-2, it has been detected only after venetoclax exposure and it can be observed several months prior to clinical relapse (~25) [103,104]. The same authors published in another small study that a median of 3 other BCL-2 mutations (different than G101V) may appear in most of R/R patients (91%) [105]. Hence, BCL-2 mutations might also act as biomarkers for venetoclax resistance.

### 3.3. Something Borrowed: Biomarkers That Retain Predictive Value on Current Treatment Algorithms

#### 3.3.1. TP53 Abnormalities

*TP53* abnormalities (TP53a) (del(17p), *TP53* mutations or both) result in a loss of p53 activity, leading to an impaired regulation of DNA repair mechanisms and resistance to chemotherapy. These abnormalities are infrequent among untreated patients (5–12%) but increase significantly during disease evolution, especially after treatment or at the time of transformation (40–60%). CIT offers no benefit to patients with TP53a that obtain very short PFS and OS regardless of the combination used [106]. Fortunately, Tas have significantly improved clinical outcomes in patients with TP53a, since they act independently of the p53 protein, which has placed them as the gold standard approach for this subset of patients. However, none of them have completely overcome the bad prognosis that TP53a carries. In the R/R setting, ibrutinib trials, with a prolonged follow-up time, showed that patients with TP53a had a shorter PFS than patients without these abnormalities (41 months vs. 57 months at RESONATE trial) [6]. Conversely, the results from a phase 2 trial (NCT01500733) that included 34 previously untreated patients with TP53a showed a median PFS and OS of 61% and 79% respectively with a follow-up time of 6.5 years [107]. These results need to be interpreted cautiously due to the low proportion of patients included in the study and the lack of randomization. In addition, the median age of the patients was 62.5 year old, suggesting that the study included a younger and highly selected population and therefore not representative. Acalabrutinib behaves very similar to ibrutinib for patients with TP53a not only in the first line but also in the R/R setting, but studies with this compound need a longer follow-up to see the long-term effect [11,12]. Venetoclax-based regimens have also demonstrated to be very active on patients with TP53a. Nevertheless, the 4-year update of the MURANO trial showed a higher proportion of patients with detectable MRD after end of trial and a trend to a lower PFS on patients carrying del(17p) without statistical significance after multivariable adjustment. In addition, the 5-year update recently communicated at the American Society of Hematology meeting confirmed that the four patients with del(17p) that achieved uMRD after end of treatment progressed [108]. Similarly, patients with TP53a from the CLL-14 trial had a shorter PFS (2-year PFS around 70%) than those with intact *TP53*. A slightly higher 2-year PFS (75–80%) has been reported on ibrutinib trials what has led some experts to recommend ibrutinib over venetoclax in TP53a [2,10]. Shortly, ongoing trials comparing both molecules will establish the preferential option for these patients. Meanwhile, we can establish that TP53a have a predictive value for a shorter duration of response to Tas especially in the context of relapse and poor response to CIT.

#### 3.3.2. IGHV Mutational Status

IGHV mutational status is a robust prognostic biomarker in CLL. M-CLL patients exhibit a more benign course of the disease with a prolonged TTFT and OS, in contrast to U-CLL [23,24,60,66,109]. Furthermore, it is also considered a valuable predictive biomarker for CIT response, especially for the FCR combination. Three independent studies demonstrated that FCR has probably the potential to “cure” a subset of patients with M-CLL. In these three studies, the PFS and OS curves reached a plateau with times comparable to age-matched healthy population [87,88,110]. However, the predictive value of IGHV mutation status has changed with the advent of Tas. These drugs achieve similar PFS and OS rates regardless of IGHV mutation status [6,10,11,12,31,111]. In addition, studies comparing Tas vs. CIT on previously untreated elderly/unfit patients (ALLIANCE A041202, CLL-14) support the preferential use of Tas regardless of IGHV mutation status [7,10]. On the other hand, the E1912 trial could possibly help to answer one of the burning questions today [8]: Will young M-CLL patients benefit more from FCR or Tas? If the answer is Tas, IGHV mutation could lose its predictive value, since it will no longer be used as a premise for first line treatment decisions. If the answer is FCR, IGHV might remain as a predictive biomarker favoring FCR for most of the M-CLL patients.

### 3.4. Something Blue: Biomarkers with Potential Predictive Value Not Fully Validated

#### 3.4.1. Complex Karyotype

The frequency of complex karyotype (CK) varies between 10% (treatment-naïve) and 40% (R/R) and is back in the spotlight again thanks to CpG-stimulation techniques [112]. Recent studies have pointed out that CK might also be identified using genomic arrays. The detection of ≥5 copy number alterations by this technique identified a subgroup of patients with independent adverse prognosis in a multicenter retrospective study that included more than 2000 patients [113].

In spite of the controversies that surround this biomarker, it seems clear that, altogether, it confers a dismal prognosis to CLL, and some guidelines advocate for its incorporation in clinical practice [3]. In the first place, there is an urgent need to generate an international standardized consensus on the exact definition of CK and how to count and interpret chromosomal abnormalities. In general, CK in CLL is defined by the presence of ≥3 abnormalities and high risk CK by the presence of ≥5 aberrations. However, not all cases with more than three abnormalities behave the same way. For instance, the association of trisomy 12 (+12), trisomy 19 and other trisomies provides good prognosis, while unbalanced translocations appear to carry a worse outcome than balanced translocations [114,115].

Secondly, CK was designated as a poor prognostic factor and a predictive biomarker for poor response to CIT based on large retrospective cohort studies [28,77,116,117]. This bad outcome has not been verified in the context of prospective randomized trials. The results of the only two trials in which CK independently predicted OS and PFS for patients treated with either FCR or chlorambucil (Chl) are illustrated in Table S2. As shown in the table, the methodology used in both studies was suboptimal.

Third and last, Tas might overcome the poor prognosis that CK entails, but this is another topic of debate. Table 1 summarizes the studies that have assessed the impact of CK in patients receiving Tas. As shown, the results are discordant.

Starting with ibrutinib, overall, treatment naïve patients that receive this drug appear to get prolonged responses irrespectively of cytogenetics [7]. On the other hand, if CK appears as a consequence of treatment, the situation changes. Even though the RESONATE trial (R/R), with a follow up of 6 years, could not identify CK as a predictive biomarker [6], other studies have linked CK to relapse, transformation and even a resistance mechanism [91,119]. In this setting CK usually comes with other dismal factors such as del(17p), del(18p) or acquired mutations, maybe acting as a confounding factor. Not surprisingly, the same conclusions can be extrapolated from acalabrutinib studies, albeit with a shorter follow-up (Table 1) [11,12,124]. With regard to venetoclax, a recent report from the CLL-14 highlights that venetoclax-obinutuzumab is able to overcome the deleterious effect of CK, in line with a previous study from real life [121,122]. Both studies need a prolonged follow-up to confirm these outcomes. In contrast, evidence from the MURANO trial (R/R) and early studies with venetoclax, showed the opposite [123] (Table 1).

Interestingly, two different studies (retrospective single center analysis with ibrutinib and the MURANO trial) have recently demonstrated that increasing karyotype complexity to ≥5 abnormalities predicts inferior survival for patients treated with TAs, similarly to what happens with CIT [31,125]. This reinforces the need for harmonization and standardization of CK definitions.

Finally, although the widespread use of idelalisib has been limited by toxicity, it is worth mentioning that this molecule might vanquish the bad prognosis of CK even for R/R patients, although with little evidence [126]. To sum up, the predictive value of CK in the era of Tas is not clear. From our point of view, patients with this biomarker should be preferentially treated with Tas, turning this marker into a predictor of better response to Tas than CIT.

#### 3.4.2. NOTCH1 Mutations

*NOTCH1* mutations are the most common recurrent mutations, seen in about 10% of CLL-patients, with increasing frequency as the disease progresses. This biomarker has been proposed as a mild negative prognostic factor, based on evidence from retrospective studies and clinical trials [73,127,128,129,130,131]. Indeed, patients with *NOTCH1* mutations constitute a heterogeneous subgroup of patients in which other factors are also important. For example the association of *NOTCH1* mutation to +12 seems to overshadow the clinical outcome of patients with +12 [132,133]. In addition, clonal and subclonal *NOTCH1* mutations predicted inferior TTFT while only clonal mutations predicted inferior OS in a cohort of patients treated with CIT [134]. Mutations of the *NOTCH1* appear to be a predictive biomarker of response to anti-CD20 treatments. The CLL8 trial showed lack of benefit from adding rituximab to conventional CT [84]. Furthermore, in trials using ofatumumab as a comparator arm, patients with wild-type *NOTCH1* performed better than those with the mutation [29]. In contrast, patients with *NOTCH1* mutations treated with obinutuzumab had a better outcome than patients treated with rituximab [135]. However, these observations need further validation to be incorporated into routine practice. In general, the PFS and OS of patients treated with Tas do not appear to be influenced by the presence or absence of *NOTCH1* mutations [29,136]. Therefore, *NOTCH1* is proposed as a biomarker that predicts inferior response to rituximab or ofatumumab, but at the moment it has no impact “by itself” in patients treated with Tas.

## 4. Prognostic Models. Different Models for Different Moments: Do Not Compare Apples with Oranges

After the enormous advance in deciphering the biological landscape of CLL, which has been accompanied by a “treatment-revolution” and a significant improve on survival, prognostic models have not evolved at the same speed [137]. Rai [33] and Binet [32] clinical staging systems, available for almost 50 years, have the merit of being in force today. Their major limitation is that none of the models is accurate enough to predict clinical outcome at the individual level and discriminate patients with early-stage disease that progress in a short period of time. With the aim of improving the accuracy of these models, many scoring systems have emerged, integrating biological and clinical variables. Most of the models were initially developed to predict OS in the CIT era. Others were designed to predict TTFT and are not influenced by treatment decisions. A recent meta-analysis published by the Cochrane Library has analyzed this topic in depth. They identified 52 prognostic models, but only 12 were externally validated (5 for TTFT; 6 for OS and 1 for PFS). Thus, prognostic models validated in the CIT period will not be the subject of this review. In this section, we review current evidence about prognostic models based on their applicability to predict TTFT and for patients treated with Tas [138].

### 4.1. Apples: Scoring Systems That Predict Time to First Treatment

The percentage of CLL patients diagnosed at early stages (Binet A, Rai 0) is now very high (~80%) due to the generalization of routine blood tests in asymptomatic people [139]. Prognostic models developed to evaluate TTFT help to counsel patients and their families, plan surveillance and identify high-risk candidates that may benefit from early intervention in the context of clinical trials. For this purpose, many scoring systems were published [36,109,140,141,142,143,144] but most of them have not been validated in independent cohorts and thus, are not transferred to clinical practice [138]. Table 2 summarizes the most relevant. Two of them deserve special mention. First, the recently published IPS-E that has been validated in nine external cohorts and only requires two clinical variables and one molecular (IGHV mutation status) for its implementation. Authors of this work concluded that the simplicity of this score might facilitate its translation to the clinic, although this requires knowledge of the IGHV mutation status since diagnosis [109]. Second, the innovative European Research Initiative on CLL (ERIC) “tailored approach” score that separates M-CLL and U-CLL and elaborates two different scores for each subset of patients. In this case, translation to the clinic will be more difficult, since it includes biomarkers not widely available such as *SF3B1* mutation or stereotyped subsets [143].

Our group recently published data comparing the accuracy of five scores (IPS-E, CLL-01, CLL-IPI, Barcelona-Brno and tailored approach) in a multicentric cohort of Binet A patients. We found that the most accurate score in predicting TTFT was IPS-E with a low concordance between the different models. In addition, none of the models was able to predict the clinical course of the disease with absolute accuracy, as one quarter of the patients could have been assigned to an incorrect risk group with any of the PIs used, underscoring that models cannot totally replace clinical expertise [146].

### 4.2. Oranges: Scoring Systems That Predict OS for Patients Treated with New Targeted Agents

As previously stated, a recent meta-analysis revealed that despite the high amount of published prognostic models, only six had been externally validated for OS and all of them were tested for patients treated with CIT. The popular CLL-IPI was the model with the best discrimination power [138]. The CLL-IPI scores the highest values to *TP53* abnormalities (4 points), B2M and U-CLL (2 points each). Given that Tas improve the poor prognosis provided by TP53a and U-CLL, it is not surprising that CLL-IPI decreases its accuracy for patients treated with these drugs. A retrospective cohort analysis of 326 frontline-ibrutinib–treated patients could analyze the CLL-IPI on 79 and found that it did not predict 12 month PFS [147]. The prognostic utility of the CLL-IPI has also been addressed in a cohort of R/R patients treated with idelalisib-rituximab in the context of phase 3 trials [148]. Most patients (~85%) were assigned to the high or very high risk subgroups and the discriminatory value of the CLL-IPI was less robust (C-statistic 0.6) than in its original publication and subsequent validation studies and meta-analyses (C-statistic 0.72). Thus, the authors proposed a modified version of the CLL-IPI, assigning only 1 point to each adverse factor and modifying the cut-off of clinical stage. An external validation study showed that this modified version of the CLL-IPI failed to provide prognostic information in a cohort of patients from real life treated with ibrutinib [149]. Molica et al. performed a systematic review that analyzed published studies that had applied the CLL-IPI to patients treated with CIT or Tas. They grouped data from the two studies previously commented and, not surprisingly, concluded that the utility of the CLL-IPI remains uncertain for patients treated with Tas [39].

Therefore, the “old” scores do not seem to be valid for patients receiving Tas. To cover this gap, novel prognostic models built with data from individuals treated with Tas have emerged. Table 3 compares and summarizes the principal characteristics of these models.

Briefly, the BALL and the NIH (Ahn et al.) scores were generated with clinical trial data, while the simplified PI and the SRS were generated with real life data. The NIH and the “simplified score” are suitable for the frontline and R/R settings. However, the “simplified score” has not been validated in other cohorts. The BALL score included patients with all type of Tas and CIT, while the other scores were applied to ibrutinib-treated-patients. The NIH score was able to assign most patients that generated secondary BTK and PLCG2 acquired mutations to the high risk group. Very recently, the BALL and the NIH (Ahn et al.) models were validated in another external study and at the same time compared to the CLL-IPI model. The discriminatory ability of the two scores was better than that of CLL-IPI. The BALL score had the best discriminatory capacity with respect to OS and the NIH score with the PFS prediction. The NIH score was the only model that provided a good prediction of both PFS and OS [152].

Interestingly, all these models used parameters related to bone marrow reserve and tumor burden, widely available worldwide, which a priori suggest that its introduction into clinical practice should be easy and straightforward. The only genetic data included in one score is *TP53* mutation, which agrees with what was previously stated about the loss of the prognostic and predictive value of many other genetic factors such as IGHV mutation status or del(11q) with the arrival of Tas. However, further work is needed to explore the relevance of including novel biomarkers such as complex karyotype, uMRD and recurrent or acquired mutations in the context of new scores. Recently, a promising dynamic score named Continuous Individualized Risk Index (CIRI) has been published and applied to patients with diffuse large B cell lymphoma, CLL and breast cancer. It has been developed with the aim of predicting a personalized probability of PFS and OS over time, considering the response to treatment as a feature. In CLL (CIRI-CLL), it has been built with variables such as the CLL-IPI, MRD or the choice of therapy. Furthermore, CIRI-CLL has demonstrated to provide superior outcome prediction to current prognostic indices (CLL-IPI) with a better C-statistic value in patients treated with CIT [153].

Other challenges include determining which is the most accurate score and whether in real life they can help the clinicians select high-risk patients who will benefit from novel treatments/combinations. Probably, new technology such as machine learning will be crucial to solve all these challenges. Machine learning is able to evaluate in a non-linearity and a more complex way data from different variables. In addition, artificial technology algorithms can deal with missing data form retrospective studies form the real-world setting to improve the precision of the models [154]. Nevertheless, clinical expertise and medical judgement should be complemented and not be replaced by models [20].

## 5. Conclusions

Predicting the outcome of CLL is an important and dynamic field of research that evolves in parallel with biological and therapeutic advances. From the patient´s perspective, it provides information that can help with personal planning. It also guides clinicians toward the best therapeutic option and is an important step in personalized medicine. Novel targeted agents have changed treatment algorithms in CLL and, consequently, the role of predictive biomarkers has been questioned. Herein, we provided a critical view of the currently most controversial issues regarding the value of prognostic and predictive biomarkers as well as prognostic models for patients with CLL treated with Tas. In summary, prognostic factors from the CIT era remain valid for predicting time to first treatment, being the most important FISH abnormalities, IGHV mutational status, *TP53* abnormalities and CD49d. Achieving uMRD after treatment is an important survival biomarker for venetoclax-based regimens but not for BTKi or PI3Ki. The few predictive biomarkers validated in the CIT era do not behave the same way right now. For instance, if an old patient is going to be treated with Tas, IGHV mutational status will no longer be a predictive biomarker for treatment election. On the other hand, the classic del(11q) might be a biomarker of better response to Tas. *TP53* abnormalities seem to ameliorate but not mitigate the poor outcome that they provide when patients receive Tas instead of CIT. CK may be the most controversial biomarker in the setting of Tas providing better outcome when it is detected in treatment-naïve than in the R/R patients. Acquired BTK and BCL-2 mutations are good candidates to be used as biomarkers for treatment failure. The predictive value of other biomarkers like *NOTCH1* mutations needs further studies. Concerning prognostic models, the accuracy of the classic systems such as the CLL-IPI has been reduced for patients undergoing Tas. Thus, new models such as the BALL and the NIH (Ahn et al.) have been proposed to predict outcome for patients receiving novel agents. For patients with early stage CLL, other scores have also been published, highlighting the IPS-E prognostic model, easily applicable and widely validated. However, all the prognostic models need to be complemented with clinical expertise.

In conclusion, despite that we are moving in the right direction, there is still scope for improvement in CLL prognostication. Some biomarkers are already extinct but surely new ones will appear and others will evolve to be incorporated in the changing landscape of CLL.

## Figures and Tables

**Table 1:** Impact of complex karyotype on clinical outcome for patients treated with ibrutinib, acalabrutinib and venetoclax.

Drug	Impact of CK	Study Type	Study	N (% CK)	Population	Prognostic Impact of CK	Others	Ref.
**Ibrutinib**	CK does not impact outcome	Phase 3 CT	RESONATE	39/153 (25%)	R/R	No impact on PFS (40 vs. 44 months, NS)		[6]
		Phase 3 CT	ALLIANCE A041202	99/333 (29%)	TN	No impact on PFS		[7]
		Pooled analysis	PCYC-1102 PCYC1103	41/132 (31%)	TN & R/R	No impact on PFS and OS in MA		[118]
		Pooled analysis	RESONATE RESONATE 2 HELIOS	41/338 (12.1%)	TN & R/R	No impact on PFS or OS	Excluded del(17p)	[89]
	CK aggravates outcome	Retrospective	Cohort	21/56 (37.5%)	R/R	Independently associated with shorter PFS and OS	17/21 del(17p)	[119]
		Pooled analysis	PCYC-1102 PCYC-1109 OSU11 RESONATE	172/295 (58%)	R/R (8 TN)	Associated with disease progression or transformation		[91]
**Acalabrutinib**	CK does not impact outcome	Phase 3 CT	ASCEND	50/154 (32%)	R/R	PFS benefit for acala arm on subgroup analysis	16 m median follow up	[11]
		Phase 3 CT	ELEVATE TN	60/358 (16.7%)	TN	PFS benefit for acala arms on subgroup analysis	28 m median follow up	[12]
	CK aggravates outcome	Phase 1/2 CT		20/57 (35%)	R/R	Shorter PFS (33m vs. NR)		[120]
**Venetoclax**	CK does not impact outcome	Phase 3 CT	CLL-14	30/200 (17%)	TN	No impact on PFS or OS (not reached in CK and non-CK)		[121]
		Retrospective	Cohort	52/130 (26.8%)	R/R (2 TN)	No impact on PFS	7 m median follow up	[122]
	CK aggravates outcome	Pooled analysis	M12-175 M13-365 M13-982	16/38 (46%)	R/R	CK independently associated with PFS	23 m median follow up	[123]
		Phase 3 CT	MURANO	94/288 (36.3%)	R/R	Shorter PFS and uMRD for CK	4 year follow up	[31]

N = number of patients; CK = complex karyotype; REF = reference; CT = clinical trial; R/R = relapsed/refractory; PFS = progression free survival; TN = treatment-naïve; OS = overall survival; MA = multivariate analysis; del(17p) = 17p deletion; m = months; uMRD = undetectable minimal residual disease; acala = acalabrutinib.

**Table 2:** Selection of prognostic models validated to predict time to first treatment.

Model	Population	Stage	Biomarkers	Risk Groups	Validation
MDAC 2011 [141]	Retrospective single-center cohort	All	IGHV ms Diameter of largest palpable LN FISH (11q/17p Vs none) N involved LN sites LDH IGHV-LDH interaction	Nomogram	1 external
O-CLL1 2016 [142]	Prospective multicenter cohort	Binet A	IGHV ms Rai stage ALC B2M	3	3 external
CLL-IPI 2016 [145]	8 Ph3 multicenter clinical trials	All	IGHV ms TP53 status B2M Clinical stage Age	3	9 external
Barcelona-Brno 2017 [140]	Retrospective single-center cohort	All (83% Binet A)	IGHV ms del(17p)/del(11q)	3	7 external
Tailored approach 2019 [143]	Retrospective multicenter cohort	Binet A	M-CLL: *TP53* abn; +12; subset #2	2	2 external
			U-CLL: *TP53* abn; del(11q); gender	3	
IPS-E 2020 [109]	Multicenter retrospective cohort	Binet A	IGHV ms ALC > 15 × 10^9^/L Palpable LN	3	9 external
CLL-1 PM 2020 [144]	Ph 3 clinical trial	Binet A	IGHV ms del(11q) del(17p) B2M LDT < 12 m Age	4	No

IGHV ms = IGHV mutation status; LN = lymph node; N = number; ALC = absolute lymphocyte count; B2M = beta-2-microglobuline; +12 = trisomy 12; TP53abn = TP53 abnormalities; m = months; LDT = lymphocyte doubling time.

**Table 3:** Description of the published prognostic scores developed to predict outcomes for patients treated with targeted agents.

Characteristics	BALL [40]	NIH (Ahn et al.) [41]	Simplified PI [150]	SRS_I_ [151]
N	2475	720	346	541
Study	Retrospective multicenter pool cohort from randomized trials	Retrospective pooled cohort from randomized trials	Retrospective multicenter cohort from academic medical centers	Retrospective multicenter working group, real life patients
Treatment	Ibrutinib Idelalisib Venetoclax CIT	Ibrutinib	Ibrutinib	Ibrutinib
Population	R/R	TN and R/R	TN and R/R	R/R
Validation cohorts	1 internal 4 external	1 internal 1 external	No	1 internal 1 external
Scores	B2M ≥ 5 → 1p LDH> ULN → 1p Hb < 11F/12M → 1p TILT < 24 m → 1p	B2M ≥ 5 → 1p LDH > 250U/L → 1p *TP53*ab → 1p Prior treatment → 1p	Age ≥ 70 → 1p R/R → 1p ECOG ≥ 1 → 1p	B2M ≥ 5 → 1p LDH > ULT → 1p Hb < 11F/12M → 2p
Groups	Low (0–1)	Low (0–1)	Low (0–1)	Low (0)
	Intermediate (2–3)	Intermediate (2)	Intermediate (2)	Intermediate (1–3)
	High (4)	High (3–4)	High (3)	High (4–5)
Prediction	OS	PFS and OS	PFS and OS	OS
Accuracy	CS = 0.79 (OS)	CS = 0.69 (PFS)	AUC = 0.6 (PFS) AUC = 0.66 (OS)	CS = 0.71 (OS)
Other		BTK and PLG2 mutations detected more frequently in the high risk group		

NIH = National Institutes of Health; Simplified PI = simplified prognostic index; SRS_I_ = survival risk score ibrutinib; N = number; R/R = relapsed/refractory; TN = treatment naïve; B2M = beta2microglobulin; ULN = upper normal limit; Hb = hemoglobin; F = female; M = male; TP53ab = TP53abnormalities; TILT = time from initiation of last therapy; ECOG = Eastern Cooperative Oncology Group; CS = C-statistic; AUC = area under the curve.

## Supplementary Materials

The following are available online at https://www.mdpi.com/article/10.3390/cancers13081782/s1, Table S1: Characteristics of main clinical trials that involve chemoimmunotherapy and oral targeted agents, Table S2: Clinical trials in which the prognostic value of complex karyotype has been evaluated for patients treated with chemoimmunotherapy.
