# Supplementary material for: From Biomarkers to Models in the Changing Landscape of Chronic Lymphocytic Leukemia: Evolve or Become Extinct
Source: Cancers (Basel). 2021 Apr 8;13(8):1782. doi: 10.3390/cancers13081782 (PMC8068228; doi:10.3390/cancers13081782)
Supplement: Supplementary file 1 [file cancers-13-01782-s001.pdf]

cancers

MDPI

Supplementary Macterial: From Biomarkers to Models in the Changing Landscape of Chronic Lymphocytic Leukemia: Evolve or Become Extinct

Isabel González-Gascón-y-Marín, Carolina Muñoz-Novas, Ana-Eugenia Rodríguez-Vicente, Miguel Quijada-Álamo, María Hernández-Sánchez, Claudia Pérez-Carretero, Victoria Ramos-Ascanio and José-Ángel Hernández-Rivas

Table S1. Names and Characteristics of main clinical trials that involve chemoimmunotherapy and oral targeted agents.

Treatment	Trial Name	Phase	Arms	N	Disease Status
	RESONATE	3	Ibrutinib	195	R/R
			Ofatumumab	196	
	PCYC-1102	1b/2	Ibrutinib	132	TN and R/R
	PCYC-1103	2	Ibrutinib	180	TN and R/R
	NCT01500733	2	Ibrutinib	34	TN and del(17p)
	PCYC-1109	1b/2	Ibrutinib+ofatumumab	71	R/R
	RESONATE-2	3	Ibrutinib	135	TN
			Chl	133	
**Ibrutinib**	HELIOS	3	BR+ibrutinib	289	R/R
			BR+placebo	289	
	RESONATE-17	2	Ibrutinib	144	R/R and del (17p)
	ALLIANCE A041202	3	Ibrutinib	182	TN
			Ibrutinib+rituximab	182	
			BR	183	
	ILLUMINATE	3	Obi+ibrutinib	113	TN
			Obi+chl	116	
	ECOG-ACRIN E1912	3	Ibrutinib+rituximab	354	TN
			FCR	175	
	CLL-8	3	FCR	408	TN
			FC	409	
	CLL-10	3	FCR	282	TN
**CIT**			BR	279	
	CLL-11	3	Obi+chl	333	TN
			Rituximab+chl	330	
			Chl	116	
	MURANO	3	Venetoclax+rituximab	195	R/R
**Venetoclax**			BR	194	
	CLL14	3	Ob+venetoclax	216	TN
			Obi+chl	216	
	ASCEND	3	Acalabrutinib	155	R/R
			Idelalisib+rituximab	155	
**Acalabrutinib**			BR	155	
	ELEVATE	3	Acalabrutinib	179	TN
			Acalabrutinib+obi	179	
			Obi+chl	177	

R/R = relapsed/refractory; TN = treatment naïve; del(17p) = 17p deletion; BR = bendamustina-rituximab; Obi = obinutuzumab; Chl = chlorambucil; FCR = fludarabine-cyclophosphamide-rituximab; FC = fludarabina-cyclophosphamide.

Table S2. Clinical trials in which the prognostic value of complex karyotype has been evaluated for patients treated with chemoimmunotherapy.

**Treatment**	**Chl +/− AntiCD20**	**FCR**
Study	CLL-11, Phase 3 trial	Phase 2 trial
Arms	Chl Vs R-Chl Vs Obi-Chl	FCR
Patients	TN	R/R
CK definition	≥3 abnormalities	≥2 abnormalities, without del (17p)
Methodology	Retrospective sample analysis from CLL-11, CpG stimulation	Conventional karyotype without CpG-stimuation
N (%) with CK	30/161 (19.5%)	22/177 (12%)
PFS	No difference in PFS	Inferior for CK in univariate and multivariate analysis (HR 2.6, $p < 0.001$)
OS	Inferior for CK in univariate and multivariate analysis (HR 2.6, $p = 0.004$)	Inferior for CK in univariate and multivariate analysis (HR 1.9, $p = 0.02$)
Reference	Herling et al. Blood, 2016	Badaux et al. Blood, 2011

Chl = chlorambucil; FCR = fludarabine, cychophosphamide, rituximab; TN = treatment naïve; R/R = relapsed/refractory; CK = complex karyotype; N = number; PFS = progression free survival; PS = overall survival; HR = hazard ratio.
